# Femoral-tibial-synostosis in a child with Roberts syndrome (Pseudothalidomide): a case report

**DOI:** 10.1186/1757-1626-1-109

**Published:** 2008-08-18

**Authors:** Ali Al Kaissi, Robert Csepan, Klaus Klaushofer, Franz Grill

**Affiliations:** 1 Ludwig-Boltzmann Institute of Osteology at the Hanusch Hospital of WGKK and AUVA Trauma Centre Meidling, 4th Medical Department, Hanusch Hospital, Vienna, Austria; 2 Orthopaedic Hospital of Speising, Paediatric Department, Vienna, Austria

## Abstract

**Background:**

Roberts syndrome (Pseudothalidomide) is a rare birth defect that causes severe bone malformation complex. The bones of the arms, and in some cases other appendages, may be extremely shortened and even absent. The fingers of the hands may be fused. An extreme case results in the absence of the upper bones of both the arms and legs so that the hands and feet appear attached directly to the body. This is called tetraphocomelia.

**Case presentation:**

We report on a two-year-old boy of Austrian origin who manifests a constellation of malformation complex include prenatal and postnatal growth retardation, craniofacial anomalies and defective development of all four extremities. The overall clinico-radiographic features were compatible with Roberts syndrome (Pseudothalidomide). Significant unilateral femoral-tibial synostosis was additional malformation.

**Conclusion:**

Associated malformations and symptoms may be the key factor in the differential diagnosis of neonatal malformation complex. Roberts's syndrome may be genetically transmitted within families as an autosomal recessive trait or may be the result of spontaneous/sporadic changes in the gene. Because the signs of the disorder so closely mimic those caused by the ingestion of thalidomide, the term "pseudo-thalidomide" is frequently used.

In this report we describe total femorotibial fusion in a child manifesting the phenotypic features consistent with Roberts syndrome from a healthy parents but first cousins in Austria. Aggressive medical intervention is of prime importance, as is forthright parental counselling when discussing the possible outcome for these patients.

## Background

The main clinical features in patients with Roberts sydrome (pseudothalidomide)are severe shortening of the limbs, with radial defects and oligodactyly or syndactyly, and a characteristic face with hypertelorism, severe cleft lip, a prominent premaxilla, a mid-face capillary haemangioma, cloudy corneae or cataracts and dysplastic or small ears. Other defects may be seen such as large genitalia, congenital heart defects and cystic kidneys [1-4]. Opitz and Lowry [5] stated that early on they had the impression that the Roberts syndrome and the SC pseudothalidomide syndrome may occur in different members of the same sibships and that the identification of identical cytological markers complements the conclusion of nosologic identity as well as effective prenatal diagnosis. Mutations in both Roberts and SC phocomelia were reported by Schule et al. [6]. They found no obvious phenotype-genotype correlation.

## Case presentation

A two-year-old child was referred to the department of orthopaedics to asssess his multiple malformation complex. The mother was a 27-year old primigarvida woman married to a31-year-old related man (first cousins). Parents did not report any history of congenital abnormalities in the family. Pregnancy history was negative for exposure to drugs or teratogens. At birth the growth parameters were below the 4 th pecentile. A characteristic dysmorphic facies associated with severe defective development of all four extremities were the major malformation complex. Cleft lip and palate was repaired when the child was 10 weeks old.

**Figure 1:**
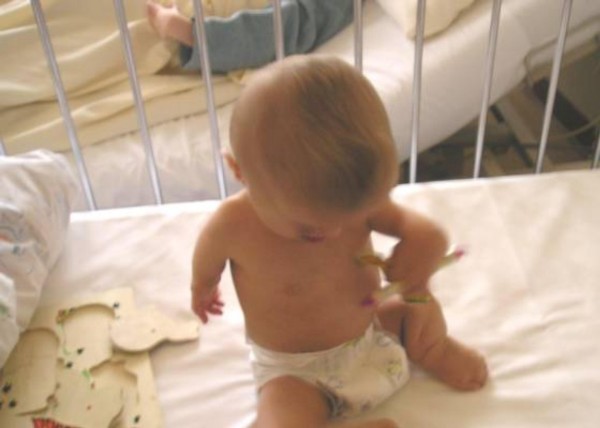
Craniofacial abnormalities such as sparse silvery blond hair, prominent frontal bones, hypertelorism, shallow orbits, prominent eyes, a median cleft lip and palate, micrognathia, hypoplastic alae nasi, small and low set ears associated with severe bone defects.

Dysmorphology examination showed a child with prenatal and postnatal growth retardation. Craniofacial abnormalities such as sparse silvery blond hair, prominent frontal bones, hypertelorism, shallow orbits, prominent eyes, a median cleft lip and palate, micrognathia, hypoplastic alae nasi, small and low set ears associated with severe bone malformation defects (figure 1).

Severe and fixed flexion deformities of the knees were evident with maximal deformity being encountered over the left knee in connection with femoral-tibial synostosis. Cytogenetic studies were normal and no premature centromere separation has been encountered. A normal 46, XY karyotype was demonstrated, and autosomal recessive inheritance was presumed on the basis of parental consanguinity. Echocardiodoppler and abdominal ultrasound were normal.

**Figure 2:**
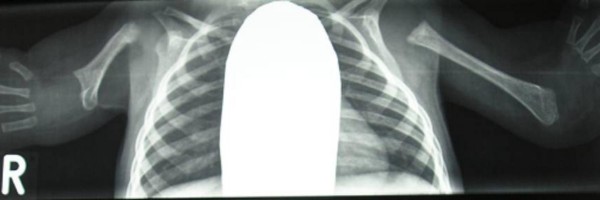
Radiographic examination showed, hypoplastic humeri, agenesis of the radii and ulnae, defective ossification of the carpals and hypoplasia of the distal phalanges.

Radiographic examination showed, hypoplastic humeri, agenesis of the radii and ulnae, defective ossification of the carpals and hypoplasia of the distal phalanges (figure 2).

**Figure 3:**
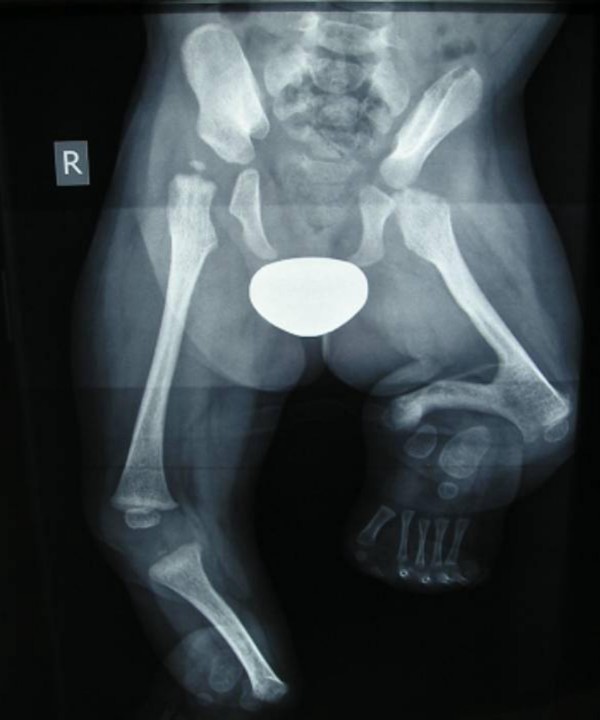
**Radiographic examination of the pelvis and the lower limbs showed hypoplastic iliac bones, a hypoplastic left femur with subsequent development of femoral-tibial synostosis.** Fixed flexion deformities of the knees was apparent, but with maximal intensity over the left knee. Bilateral fibular aplasia, the ankles showed defective ossification associated with hypoplasia of the distal phalange.

The pelvis and the lower limbs showed hypoplastic iliac bones, a hypoplastic left femur with subsequent development of femoral-tibial synostosis. Fixed flexion deformities of the knees was apparent, but with maximal intensity over the left knee. Bilateral fibular aplasia, the ankles showed defective ossification associated with hypoplasia of the distal phalanges (figure 3). His subsequent course of developmental milestones has been of severe retardation, but his mental growth was nearly normal. His primary obstacle to improve his motor development was the severe fixed flexion deformity of the left knee. The planned surgery will involve splitting of the quadriceps, exposure and then excision of the synostosis.

## Discussion

Typically the symptoms of phocomelia syndromes are undeveloped limbs and absent pelvic bones; however, various abnormalities can occur to the limbs and bones. Usually the upper limbs are not fully formed and sections of the "hands and arms may be missing." Short arm bones, fused fingers, and missing thumbs will often occur. Legs and feet are also affected similarly to that of the arms in hands. Individuals with phocomelia will often experience missing thigh bones, and the hands or feet may be of an unordinary petite size or appear as stumps due to their close "attachment to the body. There were several and different clinical entities of children born with phocomelia syndrome [1,2,6-8].

The Roberts (pseudothalidomide syndrome) is a rare autosomal recessive disorder. Roberts initially described it in 1919 [1]. It is characterised by pre and postnatal growth deficiency, symmetric limb reductions of variable severity and craniofacial anomalies including hypertelorism, hypoplastic nasal alae, cleft lip and palate. About half of the reported cases presented chromosomal abnormalities. Roberts syndrome is probably the same as SC-phocomelia in view of the fact that in both conditions chromosome analysis shows premature centromere separation. However, not all cases show this feature. Hermann et al., [2] published a family with surname begening with S and another with surname begening with C. He descibed a very similar entity called pseudothalidomide or SC syndrome in 1969.

Allingham-Hawkins and Tomkins [3] review heterogeneity in the condition. Patients with increased premature centromere separation also have cellular hypersensitivity to mitomycin C and there is evidence for different complementation groups. SC are the initial of the two families that were originally described [2]. Thalidomide primarily prescribed as a sedative or hypnotic. In Germany, between 5000 and 7000 infants were born with the qualities of Phocomelia. Thalidomide became effectively linked to death or severe disabilities among babies [8].

Previous reports described variable association of malformation complex in connection with Roberts syndrome. Satar et al., [9] reported a case with an accessory spleen and rudimentary gallbladder. Fryns et al., [10] described 2 sibs with tetraphocomelia typical of Roberts syndrome: there was almost complete reduction of the midparts of the upper and lower limbs, and characteristic oligodactyly with absent nails. Neither cleft lip/cleft palate nor eye anomalies were present. Furthermore, premature centromere separation was not observed. The facies was unusual, consisting of a beaked nose, short philtrum, and triangular mouth. Occasional cases can have craniosynostosis, causing confusion with Baller-Gerold syndrome [11]. Many affected infants die in the newborn period, and survivors may have mental retardation (although intelligence can also be normal, [12]. Femoral-tibial synostosis was not a feature. Eylon et al., [13] described femoral-tibial ankylosis in a 9.5-year-old girl who presented with Roberts syndrome.

Defective development of the limbs and femoral-tibial synostosis is a feature in connection with thrombocytopenia-absent radii (TAR) syndrome, and Schinzel phocomelia syndrome [14,15].

## Conclusion

This case report emphasizes the importance of recognizing infants born with phocomelia syndrome. Differentiating infants with Roberts-SC phocomelia from other multiple malformation syndromes that feature intercalary limb defects, including thalidomide embryopathy, TAR, and Schinzel phocomelia is fundamental.

## Abbreviations

SC-Syndrome: SC is the initial of the two families that were originally described; TAR: Thrombocytopenia-absent radii syndrome.

## Consent

Written informed consent was obtained from the parents for the purpose of publication of the manuscript and figures of their child. A copy of the written consent is available for review by the editor-in-Chief of this journal.

## Competing interests

The authors declare that they have no competing interests.

## Authors' contributions

All of the authors were involved in the clinico-radiographic assessment and finalising the paper. All authors have red and approved the final version of the paper.
